# Sex-Biased Gene Expression and Dosage Compensation on the *Artemia franciscana* Z-Chromosome

**DOI:** 10.1093/gbe/evz053

**Published:** 2019-03-13

**Authors:** Ann Kathrin Huylmans, Melissa A Toups, Ariana Macon, William J Gammerdinger, Beatriz Vicoso

**Affiliations:** Institute of Science and Technology Austria, Klosterneuburg, Austria

**Keywords:** sex chromosome, gene expression, sexual dimorphism, dosage compensation, crustacean

## Abstract

Males and females of *Artemia franciscana*, a crustacean commonly used in the aquarium trade, are highly dimorphic. Sex is determined by a pair of ZW chromosomes, but the nature and extent of differentiation of these chromosomes is unknown. Here, we characterize the Z chromosome by detecting genomic regions that show lower genomic coverage in female than in male samples, and regions that harbor an excess of female-specific SNPs. We detect many Z-specific genes, which no longer have homologs on the W, but also Z-linked genes that appear to have diverged very recently from their existing W-linked homolog. We assess patterns of male and female expression in two tissues with extensive morphological dimorphism, gonads, and heads. In agreement with their morphology, sex-biased expression is common in both tissues. Interestingly, the Z chromosome is not enriched for sex-biased genes, and seems to in fact have a mechanism of dosage compensation that leads to equal expression in males and in females. Both of these patterns are contrary to most ZW systems studied so far, making *A. franciscana* an excellent model for investigating the interplay between the evolution of sexual dimorphism and dosage compensation, as well as Z chromosome evolution in general.

## Introduction

In many species, sex is determined by a pair of sex chromosomes: X and Y chromosomes in male-heterogametic species, or Z and W in female-heterogametic species. Sex chromosomes originate from normal pairs of autosomes, but become differentiated after the sex-specific chromosome (Y or W) stops recombining (Bergero and Charlesworth 2009; Bachtrog 2013) and eventually degenerates. Much of our understanding of how this occurs was shaped by the stable XY pairs of model organisms (e.g., fruit flies and mammals), but recent studies in nonmodel organisms have uncovered and characterized a large variety of sex-determining systems (reviewed in Bachtrog et al. 2014). ZW chromosomes have now been analyzed at the genomic and transcriptomic level in several independent clades (e.g., birds, snakes, Lepidoptera, and fish), and these studies have highlighted peculiarities in the biology and evolution of these chromosomes (Ellegren et al. 2007; Itoh et al. 2007; Ellegren 2011; Vicoso and Bachtrog 2011; Walters and Hardcastle 2011; Harrison et al. 2012; Mank 2012; Vicoso et al. 2013; [Bibr evz053-B10][Bibr evz053-B41]; Ellegren 2011). In particular, while “dosage compensation” mechanisms that regulate gene expression of the X/Z to make up for loss of Y/W-linked genes are widespread in male-heterogametic species, they appear to be limited to a few dosage-sensitive genes in female-heterogametic species (with the exception of Lepidoptera, reviewed in Gu and Walters 2017 and Mank 2013). Why this should be the case, and what evolutionary forces are at play, is still unclear (Mank et al. 2011; Gu and Walters 2017).

A related topic that has been the focus of extensive research is the evolution of sexually dimorphic gene expression (Ellegren and Parsch 2007; Parsch and Ellegren 2013; Grath and Parsch 2016). Many genes are expressed at higher levels in one of the sexes, that is, they are “sex-biased.” Despite the high prevalence of sex-biased expression in virtually all species that have been studied, it is not yet clear what drives the evolution of these sex-specific expression patterns, and how these relate to morphological dimorphism (Parsch and Ellegren 2013). This question is particularly challenging for genes on sex-chromosomes, which differ in copy number between the sexes, and can differ in the extent to which they are compensated (Parsch and Ellegren 2013; Grath and Parsch 2016), but are also predicted to accumulate an excess of mutations/genes with sex-specific benefits (Rice 1984). Disentangling whether sex-biased expression of sex-linked genes is due to a lack of equalization or to the accumulation of genes with sex-specific functions can be difficult (Parsch and Ellegren 2013; Huylmans et al. 2017).

Crustaceans provide an excellent independent group to address these questions, as they have almost all sex-determining systems: genetic (with XY and ZW sex chromosomes), polygenic, environmental, and even parasitic cytoplasmatic sex determination systems can be found in this clade (Subramoniam 2016). Given their economic importance, much of the work so far has focused on physiological components of sexual differentiation, such as the central role of the androgenic gland in “higher” crustaceans (Subramoniam 2016). On the other hand, not much is known about the molecular mechanisms underlying this diversity, and about the biology and evolution of their sex chromosomes (Subramoniam 2016). For instance, there are over 20 crustacean genomes listed on NCBI (https://www.ncbi.nlm.nih.gov/genome/?term=txid6657[Organism:exp]; last accessed March 26, 2019), but these do not for the most part contain linkage information, and, aside from a few exceptions (Cui et al. 2015; Reisser et al. 2017; Baldwin-Brown et al. 2018), their sex chromosomes have not been characterized at the genomic or transcriptomic level. Consequently, no systematic assessment of expression of sex-linked genes has been performed, and it is unclear if dosage compensation occurs in this group. Finally, while homologs of known sex-determining genes of insects, including *dsx* and *tra*, have been implicated in sex determination (Kato et al. 2011; Subramoniam 2016), the master-switch of the pathway and the downstream genes are still unknown.

Here, we focus on *Artemia franciscana*, an American brine shrimp species commonly used in the aquarium trade. Like other brine shrimp, *A. franciscana* lives in hypersaline lakes that are adverse to all but a few organisms, and often vary in water levels and salinity concentrations (Gajardo and Beardmore 2012). Their reproductive cycle can either lead to the production of live offspring, when conditions are favorable, or to the production of cysts that can survive dehydration for long periods of time (Gajardo and Beardmore 2012). *Artemiafranciscana* has genetic sex determination, and a pair of ZW chromosomes has been described (Bowen 1963; Parraguez et al. 2009). Although a genetic map has found a genomic region fully linked to females (and therefore W-linked) (De Vos et al. 2013), there is inconsistency in the extent of ZW differentiation that has been detected, with one cytological study showing heteromorphic sex chromosomes (Parraguez et al. 2009), and another failing to find morphological differences between the Z and the W (Accioly et al. 2015). While the gene that plays the role of sex determination master-switch is unknown, two genes have been suggested to be involved in sex determination. First, a homolog of *DMRT* has been shown to have sex-specific expression in the gonads of several *Artemia* species (Farazmand et al. 2010). Second, a homolog of the Lepidoptera sex-determining gene *Masculinizer* (*Masc*) (Kiuchi et al. 2014) was shown through RNAi downregulation to be part of the pathway that controls sexual differentiation (Li et al. 2017). The connection between the (putative) sex-linked master switch of sex determination and these genes is at this point unknown.

Finally, an earlier study compared a male and female transcriptome (Valenzuela-Miranda et al. 2014), and found extensive sex-specificity of the transcript content and gene expression patterns. However, only pooled whole animals were used, and it is unclear which tissue these differences arose from. This is important because in this species, heads, despite being somatic, show extensive morphological dimorphism: males have enlarged second antennae that function as claspers. This trait is likely to have been driven by sexual selection, since claspers are used for mate guarding before and after mating (Rode et al. 2011). *Artemia* therefore provide an unusual opportunity to understand the evolution of genes expressed in a somatic tissue with extensive differentiation and evolving under sexual selection. Another limitation was that Z-linked and autosomal genes were not analyzed separately, which could potentially bias the results if dosage compensation is incomplete.

In this study, we first characterize gene expression in male and female heads and gonads, giving us a tissue-specific overview of sex-biased expression. We further obtain genomic reads from males and females, and combine them with a publicly available genome to detect genes that are on the Z chromosome. Our results provide a first overview of the sex-linked genes of this species and of their expression in males and females, and allow us to test whether mechanisms of dosage compensation are globally balancing the expression of Z-linked genes.

## Materials and Methods

### Specimen Obtention and RNA and DNA Sequencing

Vacuum-packed *A. franciscana* cysts (from Great Salt Lake, USA) were purchased from Sanders (Utah, USA), and hatched at 25 °C, in 27 g/l salinity, with gentle aeration and under constant fluorescent light. Nauplii were maintained at 30 g/l salinity, 14 h:10 h day:night cycle until adulthood. Adults were kept at 60 g/l salinity. From day 28, individuals were kept in individual containers to prevent mating. Male and female adults were dissected and total RNA was extracted from pools of 5 gonads or 5 heads using the Bioline Isolate II RNA Mini Kit (Cat. No. BIO-52073). RNA-sequencing (RNA-seq) with two replicates per sex and tissue was performed on an Illumina HiSeqV4 producing 125-bp paired-end (PE) reads.

An inbred iso-female line was further produced by six generations of sib–sib mating (which should lead to an 80% reduction in heterozygosity, and greatly reduce the number of copy number variants that could erroneously be detected as sex-linked). DNA was extracted from a whole male and a whole female of this inbred line using the Qiagen DNeasy Blood & Tissue Kit (Cat. No. 69506). Library preparation and 100-bp PE sequencing were performed at the Vienna Biocenter Next Generation Sequencing (NGS) Core Facility. All reads have been deposited in the NCBI Short Reads Archive under project accession number PRJNA524488, and the transcriptome is available at the IST Data repository (URL: https://doi.org/10.15479/AT:ISTA:6060; last accessed March 26, 2019).

### Transcriptome Assembly and Estimation of Gene Expression

Transcriptome assemblies were performed using all eight RNA-seq libraries. First, sequencing libraries were quality checked using FastQC (version 0.11.5; Andrews 2010) and subsequently, reads were trimmed using Trimmomatic (version 0.36; Bolger et al. 2014), retaining only reads with a minimum of 90 bp after trimming. This resulted in a total of 472 million reads that were used for the de novo transcriptome assemblies. Assemblies were performed in PE mode with the assemblers SOAPdenovo-Trans (version 1.03; Xie et al. 2014) for multiple K-mers (31–81 with a step size of 10), Trans-ABySS (version 1.5.5; Robertson et al. 2010) for multiple K-mers (40–84 with a step size of 4), and Trinity (version 2.2.0; Grabherr et al. 2011) for K-mer 25. SOAP assemblies were merged and CD-HIT-EST (version 4.7; Fu et al. 2012) was run with a sequence identity threshold of 1.0 to remove redundancy. ABySS assemblies were merged with transabyss-merge. For all three assemblers, only sequences with a minimum length of 200 bp were retained. These were then merged using the EviGene pipeline (Gilbert 2016) which resulted in an “okay” set of transcripts deemed most likely to be biologically relevant, their coding sequences (CDS), and their protein sequences (as well as a set of alternative transcripts that was not used further). This “okay” set was used for all downstream analyses.

The quality of the de novo assembled transcriptome was tested using the BUSCO pipeline (version 3; Simão et al. 2015) which assesses the presence and fragmentation of highly conserved single copy orthologs that should be present in all species. As the reference, the arthropod set of OrthoDB (version 9.1; Zdobnov et al. 2017) was chosen with *Daphnia pulex* as the most closely related species. We compared the completeness and level of duplication and fragmentation to the published *A. franciscana* transcriptome (Valenzuela-Miranda et al. 2014) and the gene annotation of the *A. franciscana* genome recently made available by the Korean Polar Institute (version 1.0, downloaded from http://antagen.kopri.re.kr/project/genome_info_iframe.php?Code=AF01; last accessed March 26, 2019).

In order to reduce the number of redundant transcripts and contaminants in our sample, we mapped all transcripts to the genome using Blat (version 35x1; Kent 2002) with a minimum match length of 100, a percent identity exceeding 95%, and a minimum percent length match of 90%. Transcripts that did not map to the genome were discarded. If two genes overlapped by >20 bp on the genome, only the transcript with the largest mapping score was kept, yielding a final set of 58,184 transcripts for further analysis. To call sex-biased genes, RNA-seq reads were mapped to the CDS set of the filtered transcriptome using NextGenMap (version 0.5.4; Sedlazeck et al. 2013). The raw counts per transcript were used to identify sex-biased genes with the Bioconductor package DESeq2 (version 1.14.1; Love et al. 2014) in R (version 3.3.3; Team 2013). Multiple testing correction was performed with the Benjamini–Hochberg correction as built into DESeq2 and genes with an adjusted *P* value <0.05 were considered significantly sex-biased. This was done separately for heads and gonads. The overlap in sex-biased genes between tissues and the numbers of sex-biased genes on the Z chromosome were tested with Fisher’s exact test.

For the dosage compensation analysis, gene expression levels were calculated as reads per kilobase of transcript per million mapped reads (RPKM) and quantile normalization was performed within each tissue. Only genes where all four samples of that tissue showed RPKM ≥ 1 were considered expressed and were used to compare overall autosome and Z chromosome expression patterns (cut-offs of RPKM ≥ 0, RPKM ≥ 2, and RPKM ≥ 5 were also tested but the results did not differ). For the chromosomal assignment (see below), only scaffolds >5 kb were used (although thresholds of 1 kb, 2 kb, and 10 kb were also tested but lead to the same results) resulting in 36,765 autosomal and 677 Z-linked transcripts in heads and 36,756 autosomal and 659 Z-linked transcripts in gonads. Replicates were averaged, and a second round of quantile normalization was performed on the averaged values. The RPKM values of these genes were compared between chromosomes and the sexes using Wilcoxon tests. To test if the results are reliable, we also used only the genes from the qPCR-confirmed Z-linked scaffolds (see below) to test for dosage compensation. This reduced the number of expressed Z-linked transcripts to 32 and 29 in heads and gonads, respectively.

### Detection of Z-Linked Genes

Cytological analyses of *Artemia* species have produced conflicting results on whether the sex chromosomes are karyotypically heteromorphic (Parraguez et al. 2009; Accioly et al. 2015). Therefore, we developed a pipeline to detect both highly diverged regions and only slightly differentiated regions of the Z chromosome.

The sequences of heteromorphic sex chromosomes are often highly diverged, and typically assemble into separate scaffolds for the Z and W. To detect these regions of high divergence, we mapped female and male genomic reads to the Korean Polar Institute genome assembly (version 1.0, http://antagen.kopri.re.kr/project/genome_info_iframe.php?Code=AF01; last accessed March 26, 2019), and assigned scaffolds based on their relative genomic coverage in the male and female samples. Raw BAM files were converted to fastq files using the “fastq” function in SAMtools (version 1.5; Li et al. 2009). Trimmomatic (version 0.36; Bolger et al. 2014) was used to remove adaptors and trim sequences in PE mode with default parameters. We aligned trimmed reads to the genome using the local alignment mode in Bowtie2 (version 2.4.3.1; Langmead and Salzberg 2012), and extracted uniquely mapped reads from the resulting SAM file. To determine the relative coverage of male and female reads, we ran soap.coverage (version 2.7.7, https://github.com/gigascience/bgi-soap2/tree/master/tools/soap.coverage/2.7.7; last accessed March 26, 2019) on each sample. Average coverage was ∼28× for the female and 30× for the male.

In order to detect Z-specific sequences, we first filtered for scaffolds with a minimum of ¼ the average coverage in the male sample and a maximum of 4× the average coverage in each sex. We computed the median log_2_(female:male) coverage and defined Z-linked scaffolds as having a log_2_(female:male) coverage between median −2 and median −0.5. Autosomal scaffolds were defined as having log_2_(female:male) coverage greater than median −0.5 but less than median +2. The cutoff of median-0.5 stems from the idea that if we have two (approximately normal) distributions of log2(coverage_female/coverage_male), one for autosomal genes centered at median-0 and one for Z-linked at −1, then using the equidistant point between the maximum of the two peaks (∼median-0.5) should yield a fairly low false positive rate for each class.

In order to test the robustness of our assessment of dosage compensation (see previous section), we performed the same expression analysis using more strictly defined coverage cutoffs for the Z chromosome and autosomes. We assessed dosage compensation using three thresholds (threshold 1: median −2 ≤ *Z* ≤ median −0.6; median −0.4 ≤ *A* ≤ median +2, threshold 2: median −2 ≤ *Z* ≤ median −0.7; median −0.3 ≤ *A* ≤ median +2, threshold 3: median −2 ≤ *Z* ≤ median −0.8; median −0.2 ≤ *A* ≤ median +2). In all three thresholds, the Z chromosome showed evidence of complete dosage compensation (supplementary fig. S2, Supplementary Material online).

Additionally, to assess our false positive rate, we did the same analysis but for scaffolds that had coverage patterns consistent with a differentiated X-chromosome (“pseudo-X-linked,” which should result from noise in the data). We filtered for scaffolds with at least a minimum of ¼ the median coverage in females and a maximum of 4× the median coverage in both sexes. We defined pseudo-X-linked scaffolds as having log_2_(female:male) coverage between median +0.5 and median +2, and autosomal scaffolds as having log_2_(female:male) coverage between median −2 and median +0.5.

In younger regions of the sex chromosomes, homologous regions of the Z and W likely assemble into a single scaffold, and are differentiated only by alleles. To detect these regions, we examined patterns of SNPs on scaffolds that were assigned as autosomal based on our coverage pipeline. First, we detected SNPs using our eight samples of RNA-seq data: 2 pools of male testes, 2 pools of female ovaries, 2 pools of male heads, and 2 pools of female heads. Reads for each pool were aligned to the genome using STAR (version 2.6.0a; Dobin et al. 2013), and processed using Picard (version 2.18.2, https://broadinstitute.github.io/picard/; last accessed March 26, 2019). We ran SAMtools mpileup (version 1.5; Li 2011) with the probabilistic alignment disabled, and called SNPs using Varscan (version 2.4.3; Koboldt et al. 2012) with a minimum variant allele frequency of 0.15 and a minimum threshold for homozygotes of 0.85, a minimum of 10 reads per site, and a Phred quality score >20. VCFtools (version 0.1.15; Danecek et al. 2011) was used to exclude indels and retain only sites with a maximum of two alleles. SNPs that were heterozygous in all female pools and homozygous in all male pools, consistent with a ZW/ZZ genotype, were extracted using custom scripts. Furthermore, since our data are pools, standard genotyping protocols likely introduce a high rate of false positives for heterozygote calls, which may inflate our ZW-consistent or XY-consistent SNP classes. To reduce this bias, we only called a site as heterozygous if the read count of the minor allele exceeded 30%. We only considered scaffolds with a minimum of 10 SNPs. We defined scaffolds as ZW-consistent if at least 20% of their SNPs were ZW-consistent. We then assessed whether the ZW-consistent scaffolds contained SNPs also found in our genomic data sets from the single male and single female used in our coverage analysis. SNPs in the DNA samples were identified using the same pipeline as used for the RNA-seq reads. In order to determine our false positive rate, we performed the same analysis for XY-consistent SNPs. Our choice of threshold for assigning scaffolds to the undifferentiated region of the Z (>20% ZW-consistent SNPs) was driven by this reverse “Young XY” analysis (supplementary fig. S4, Supplementary Material online), in which only two scaffolds were above this value.

We finally assessed whether any candidate sex-determining were located on the Z scaffolds. We downloaded all protein sequences for *Drosophila melanogaster* (version r6.18) from Flybase, and selected the longest isoform for proteins with a known function in sex determination. We then used tblastn (version 2.2.31+) to screen the *A. franciscana* genome for candidates, and selected hits with a maximum e-value of 10^−5^.

### qPCR to Verify Z-Linkage

Ten large scaffolds in the Z-linked tail of the coverage distribution [log2(Fcov/Mcov)<(−0.6)] were selected for additional validation of Z-linkage through qPCR (supplementary table S2, Supplementary Material online). Two additional putative autosomal scaffolds [log2(Fcov/Mcov)>(−0.2)] were selected as controls. The published *Masc* sequence (obtained from https://www.ncbi.nlm.nih.gov/nuccore/KY245899.1; last accessed March 26, 2019) was also tested to confirm its presence on an autosome. DNA was extracted from whole bodies of three *A. franciscana* females and three *A. franciscana* males using QIAGEN DNeasy Blood and Tissue Kit (Qiagen, Germany). One individual of each sex came from an isofemale line that had undergone 6 generations of full-sib matings and two individuals came from a lab-reared colony. DNA was quantified using Invitrogen Qubit 2.0 fluorometer (ThermoFisher Scientific, USA).

A single annotated exon was selected on each genomic scaffold (using the GBrowse Exon viewer, available at http://antagen.kopri.re.kr/project/genome_info_iframe.php?Code=AF01; last accessed March 26, 2019; the list of selected genes is provided in supplementary table S2, Supplementary Material online), and was used to develop primers for qPCR using Primer3 (Untergasser et al. 2012). Similarly to the methods outlined in Nguyen et al. (2013) and Rovatsos et al. (2014), these genomic target sequences were amplified with qPCR to detect the 2-fold copy number difference between males and females expected on a Z-specific region (Nguyen et al. 2013; Rovatsos et al. 2014). Each qPCR reaction began with 5 ng of genomic DNA, the primer pair for the target sequence and KAPA SYBR FAST qPCR Master Mix (KAPA Biosystems, South Africa). The qPCR parameters involved an initial denaturation step at 95 °C for 3 min followed by 40 cycles of PCR with a denaturation step at 95 °C for 10 s followed by an annealing and elongation step at 55 °C for 30 s. The reaction was run on a Bio-Rad C1000 Thermal Cycler with a CFX96 Real-Time System (Bio-Rad, USA) and the qPCR product was quantified using ΔΔCq. All samples in supplementary figure 1, Supplementary Material online, were normalized to the autosomal genes, *Art-11161* and *Art-15564* (these names correspond to the gene names in the publicly available genome annotation, since the primers were designed based on annotated exons). The samples in figure 4 were only normalized to *Art-15564* in order to demonstrate the expected pattern from a putative autosomal gene, *Art-11161*. Due to the high variance in primarily *Art-04698*, but also *Art-08885*, we subsequently extracted DNA using the aforementioned methods for two additional males and females from the lab-reared colony and similarly evaluated these individuals using qPCR for *Art-04698* and *Art-08885* with *Art-11161* and *Art-15564* as controls. 

## Results

### Transcriptome Assembly and Sex-Specific Expression in Gonads and Heads

**Fig. 1.**
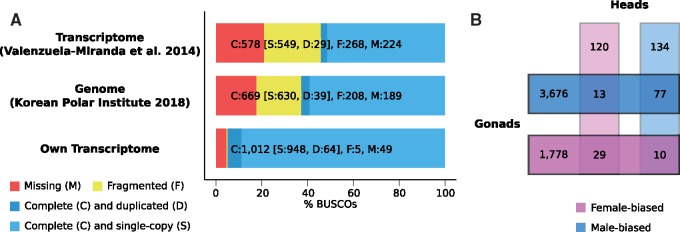
—Transcriptome quality and sex-biased genes. (*A*) Shows the quality of the published transcriptome (Valenzuela-Miranda et al. 2014), the transcripts from the genome made recently available by the Korean Polar Institute (2018), and our own transcriptome assembly based on BUSCO completeness of 1,066 highly conserved single-copy orthologs. (*B*) Shows the numbers of sex-biased genes in heads and gonads and the overlap between the tissues.

Male and female RNA-seq reads obtained from heads and gonads were pooled and assembled with multiple assemblers and K-mers (Materials and Methods). These multiple assemblies were merged, and the most biologically relevant set of transcripts was selected using the EviGene pipeline (Gilbert 2016). The final set contained 99,222 transcripts with an N50 of 1,251 bp and an average size of 743 bp, compared with 36,896 transcripts in Valenzuela-Miranda et al. (2014), and 19,631 annotated genes in the publicly available genome assembly (Unit of Polar Genomics, Korea Polar Research Institute [KOPRI], http://antagen.kopri.re.kr/project/project.php; last accessed March 26, 2019). We evaluated the completeness and quality of the three gene sets using BUSCO (Simão et al. 2015). Figure 1*A* shows that the present assembly has the highest BUSCO score and the smallest number of missing or fragmented genes, even though the large number of assembled scaffolds suggests that some redundancy or fragmentation is left even after the EviGene filtering. This improved assembly should therefore be a useful resource for future *Artemia* and crustacean research.

About 58,184 transcripts mapped nonredundantly to the genome, and were used for further gene expression analyses. Contrary to what was previously reported (Valenzuela-Miranda et al. 2014), only a few transcripts were fully sex-specific (i.e., they had an RPKM < 0.1 in the two replicates of one sex and >1 in the two replicates of the other): 43 in the head and 154 in the gonads. On the other hand, significant sex bias of expression was widespread (fig. 1*B*). The vast majority of sex-biased expression was detected in the gonads, where >1,000 transcripts had a significant female-bias and >3,000 were male-biased. However, several hundred transcripts were also sex-biased in the head (162 female-biased and 221 male-biased), consistent with the morphological dimorphism of this tissue. Finally, 29 and 77 transcripts were female- and male-biased in both tissues (many more than expected by chance, *P* value < 0.0001), and 2 were female- and 2 were male-specific in both tissues. Sex determination appears to be cell-specific in *Artemia* (Subramoniam 2016). Genes that are consistently expressed in a sex-specific manner may provide interesting new candidates, as sexually dimorphic tissues must rely directly on the expression of the sex determination cascade, which in turn must have consistent sex-specific patterns of expression.

### Detection of Z-Specific Genes

**Fig. 2.**
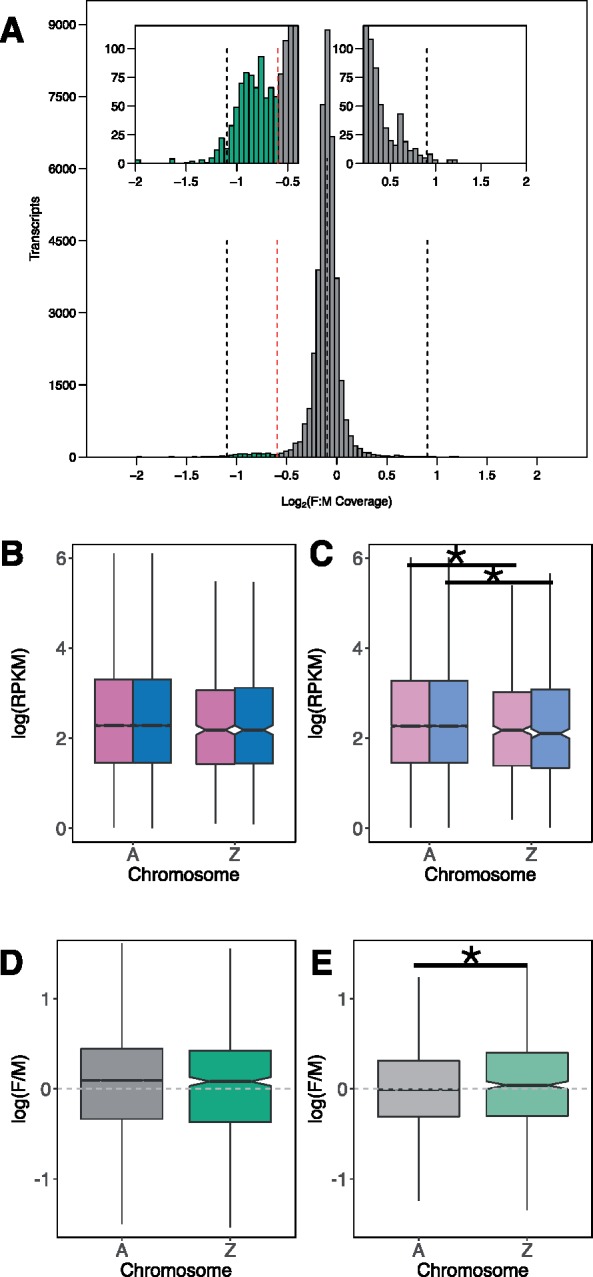
—Z chromosome identification and comparison of gene expression patterns with the autosomes. (*A*) Log_2_(Female:Male genomic coverage) of transcripts. Median log_2_(Female:Male coverage) shown as the central black dashed line. Median coverage ±1 are also shown as vertical black dashed lines. Cutoff for Z-chromosome coverage shown as dashed red line. Transcripts to the left of the dashed red line are assigned to the Z chromosome, and right of the dashed red line are assigned to autosomes. (*B*) Gene expression of females (pink) and males (blue) on the autosomes (“A”) and the Z chromosome (“Z”) in gonads. (*C*) Gene expression of females (pink) and males (blue) on the autosomes (“A”) and the Z chromosome (“Z”) in heads. (*D*) Female-over-male (“F/M”) expression ratios compared for the autosomes (“A,” gray) and the Z chromosome (“Z,” green) in gonads. (*E*) Female-over-male (“F/M”) expression ratios compared for the autosomes (“A,” gray) and the Z chromosome (“Z,” green) in heads. Wilcoxon test, *P value < 0.05, **P value < 0.01.

Previous cytological work identified morphological differences between the Z and W chromosomes, suggesting that the W in this species may be quite differentiated (Parraguez et al. 2009, but see Accioly et al. 2015). To identify the region of the Z that is highly differentiated from the W, we mapped whole genome sequencing data from a single male and a single female sample to the publicly available genome (Unit of Polar Genomics, Korea Polar Research Institute [KOPRI], http://antagen.kopri.re.kr/project/project.php; last accessed March 26, 2019; NCBI Bioproject PRJNA449186), and examined the resulting log2(female:male) coverage ratio. Median coverage in the male and female samples was similar, 30× and 28×, respectively. The median log2(female:male) ratio for scaffolds exceeding 5 kb was −0.096, therefore, we defined Z-specific scaffolds as those having a log2(female:male) ratio between median −2 and −0.596 (median −0.5; fig. 2*A*). We were able to assign 91% of the genome to the Z chromosome or to the autosomes. The differentiated part of the Z chromosome contains 696 scaffolds, totaling 19 Mb of sequence, and corresponds to 2% of the genome assembly. We then mapped our de novo transcriptome assembly to the same genomic scaffolds. The differentiated part of the Z chromosome contains 712 out of 39,667 mapped transcripts expressed with a minimum level of 1RPKM in all samples (fig. 2*A*). As a measure of our false positive rate, we examined scaffolds with a log2(female:male) ratio between median +0.5 and median +2, which is consistent with a differentiated X chromosome. This identified only 240 transcripts, suggesting the majority of Z-specific transcripts are true positives.

To further test the effectiveness of our bioinformatic pipeline, we selected ten large scaffolds to analyze for additional validation of Z-linkage using qPCR on DNA extracted from three males and three females (see Materials and Methods, and Nguyen et al. 2013 and Rovatsos et al. 2014 for validations of the approach to detect sex-linked sequences). This analysis confirmed that eight out of the ten predicted Z-linked scaffolds from the coverage analysis were in fact Z-linked (containing 42 of our de novo transcripts in total), while only two predicted Z-linked scaffolds from the coverage analysis followed a pattern consistent with what would be expected from an autosome (supplementary fig. S1, Supplementary Material online).

### Dosage Compensation throughout the Z-Specific Region

We used the chromosomal assignment of our de novo transcripts to assess the status of dosage compensation in the differentiated part of the Z-chromosome of *A. franciscana*. Comparing male and female expression levels of expressed genes (RPKM ≥ 1) on the autosomes and the Z chromosome shows that a global dosage compensation mechanism is likely at play, as there is no significant difference in the expression of Z-specific genes between the sexes in either gonads (*P* value = 0.93, fig. 2*B*) or heads (*P* value = 0.6, fig. 2*C*). Furthermore, the female-over-male expression ratio of the Z-specific region does not differ from that of the autosomes in the gonads (*P* value = 0.53, fig. 2*D*), and is in fact slightly higher in the head (*P* value = 0.01, fig. 2*E*; however, this difference disappears when different cutoffs are used to call Z-specific scaffolds, see below). In both sexes, the Z chromosome has lower expression than the autosomes in the heads (*P* value(males) = 0.004, *P* value(females) = 0.03, fig. 2*B*). This may suggest that down-regulation of Z expression in the homogametic males could play a role in dosage compensation, similar to what happens in Lepidoptera (Kiuchi et al. 2014). However, reduced expression of the Z is not observed in the gonads (*P* value(males) = 0.09, *P* value(females) = 0.1, fig. 2*C*), and without knowing how variable levels of expression are between different autosomes, it is unclear if the Z-chromosome is a true outlier.

Our definition of the Z chromosome (median −2 ≤ *Z* ≤ median −0.5) may include some autosomal scaffolds that would bias our results. In order to test the robustness of our assessment of dosage compensation, we performed the same analysis using three increasingly stringent cutoffs for the Z chromosome and autosomes (see Materials and Methods). In all three thresholds, the Z chromosome showed similar expression levels in males and females (supplementary fig. S2, Supplementary Material online).

As a second verification, we examined if this pattern of dosage compensation holds when using only transcripts found on the qPCR-confirmed Z-linked scaffolds (supplementary fig. S3, Supplementary Material online). Again, we saw no difference in the female-to-male ratio of the Z chromosome compared with the autosomes (gonads: *P* value = 0.65, supplementary fig. S3*C*, Supplementary Material online; heads: *P* value = 0.43, supplementary fig. S3*D*, Supplementary Material online), although the power of this analysis is limited by the small number of genes.

Finally, very few sex-biased genes are located on the Z chromosome (3 male-biased genes and 1 female-biased gene in heads; 45 male-biased genes and 24 female-biased genes in gonads). Sex chromosomes often show an excessive accumulation of sex-biased genes, such as the feminization of the X chromosome in *Drosophila melanogaster* and other flies (Parisi et al. 2003; Vicoso and Bachtrog 2015) or the masculinization of the Z chromosome in Lepidoptera or birds (Wright et al. 2012; Huylmans et al. 2017). Sex-biased expression is frequently interpreted as a proxy for (partially) resolved sexual conflict, which should in theory be most pronounced on the sex chromosomes (Rice 1984). Our results suggest that sex-biased expression is not necessarily a feature of well-differentiated sex chromosomes, even when gonad expression is considered, arguing against a primary role of sexual antagonism in shaping the distribution of sex-biased genes in this species.

### Evidence for a Nonrecombining but Undifferentiated ZW Region

Loss of recombination of sex chromosomes often occurs in a stepwise manner, producing “evolutionary strata” with varying degrees of divergence. We therefore searched for scaffolds consistent with regions of the sex chromosome that have diverged more recently, in which the Z and W coassemble and are undetectable using our coverage pipeline. We first searched for polymorphic sites in our eight samples of RNA sequencing from four pools of males and from four pools of females. We searched for sites consistently heterozygous in all four female samples and homozygous in all four male samples, consistent with a ZW and ZZ genotype, respectively (We required a minimum of 30% of reads to support the minor allele in order for a site to be called heterozygous). This identified 1,257 SNPs that were ZW-consistent.

**Fig. 3.**
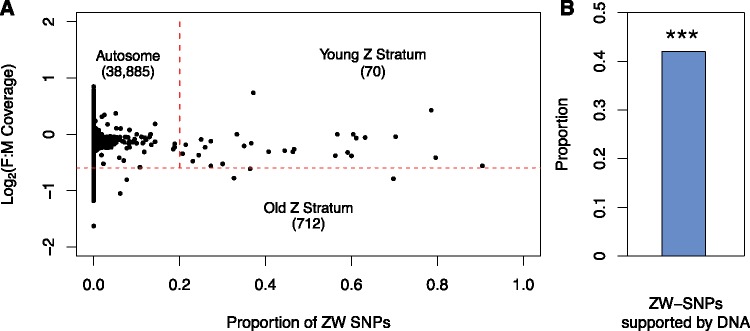
—Identification of undifferentiated ZW region. (*A*) Coverage and proportion of ZW SNPs on each scaffold. Scaffolds below the horizontal red dashed line are defined as Z-specific based on their log_2_(female:male) coverage ratio. Autosomal scaffolds are above the horizontal red dashed line and to the left of the vertical red dashed line. Young Z scaffolds are above the horizontal red dashed line and to the right of the vertical dashed line, and are defined by having >20% of the SNPs consistent with ZW-inheritance. The number of transcripts in each category is given in parentheses. (*B*) The proportion of sex-chromosome consistent SNPs in the RNA data that are also consistent in the DNA sequencing data set. Only SNPs on scaffolds with >20% of SNPs supporting a ZW karyotype in the RNA data set (putative young ZW strata) were considered. *** denotes a significant enrichment in the proportion of ZW-consistent SNPs in the RNA that were also ZW-consistent in the DNA, compared with the same proportion for all SNPs identified in the RNA (*P* < 2.2×10^−16^).

We then selected scaffolds in which at least 20% of the SNPs were ZW-consistent. This yielded 29 scaffolds containing 542 ZW-consistent SNPs. We cross-referenced these 542 sites with our genomic samples of a single male and single female. Only 110 ZW-consistent SNPs were on sites with sufficient coverage to be assessed in the male and female DNA samples. Of these SNPs, 46 (42%) were also ZW-consistent in the DNA samples (fig. 3). This value is much greater than the general percentage of RNA-detected SNPs that were ZW-consistent in the DNA (46/110: 42% vs. 5,165/145,204: 3%, χ^2^ = 454.4, *P* value < 2.2 ×10^−16^; supplementary table S1, Supplementary Material online), supporting the existence of a nonrecombining but undifferentiated ZW region.

To test our false positive rate, we reanalyzed our data using the same methodology, but for XY-consistent SNPs (which should result from noise in the data). We identified 656 XY-consistent SNPs in our RNA data set, of which 30 fell on scaffolds with an excess of 20% XY consistency (an 18-fold reduction compared with the fraction of ZW-consistent SNPs on scaffolds with >20% ZW consistency, %, χ^2^ = 303.71, *P* value < 2.2 ×10^−16^). Of the 10 that had sufficient coverage in the DNA data set, none were XY-consistent (supplementary fig. S4*D*, Supplementary Material online), further indicating that our ZW-consistent SNPs are biologically relevant, and not simply detected due to noise in the RNA and DNA data sets.

In total, we identified 1.4 Mb of sequence containing 70 transcripts on the putative undifferentiated ZW region (fig. 3*A*), much less than the fully differentiated region of the Z chromosome. However, our methods for detection of undifferentiated ZW regions are not as powerful, and a larger nonrecombining region may be detected in the future with more extensive population data.

### The Genomic Location of Candidate Sex-Determining Genes

**Fig. 4.**
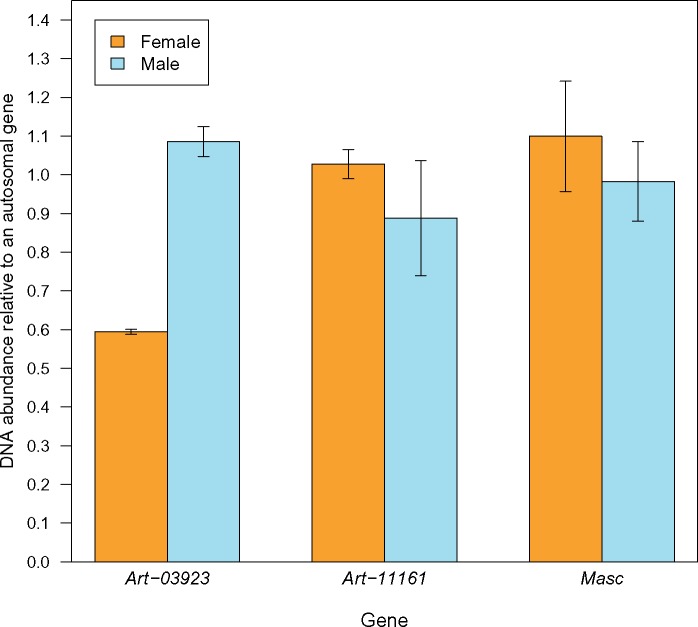
—Testing for sex-linkage of *Masc*, a gene known to be involved in sex determination. qPCR amplification results are shown for a Z-linked gene annotation (*Art-3923*), for an autosomal control (*Art-11161*), and for *Masc*.

A homolog of the *Bombyx mori* gene *Masc* has recently been implicated in sex determination in *A. franciscana* (Li et al. 2017). Similar to what happens in Lepidoptera, the *Artemia* MASC protein seems to drive the development of the male phenotype (Li et al. 2017). Downregulation of this gene using RNAi leads to a female-biased progeny, suggesting that it works in a dosage-sensitive manner. It would therefore be plausible for *Masc* to be a primary sex-determining gene if it were located on the Z chromosome, similar to the dosage-sensitive masculinizing *DMRT1* in birds (Smith et al. 2009; Graves 2014). To test for this possibility, we first mapped the published *Masc* sequence to the genomic scaffolds of *A. franciscana*. The scaffold that it maps to, scaffold169, has coverage patterns consistent with an autosomal locus (log_2_(F:M)=−0.09, well above our threshold for Z-linkage), and harbors no ZW-consistent SNPs. We further designed primers using a single exon of *Masc* and used qPCR on DNA extracted from three males and three females to test for copy number differences between the sexes (fig. 4). Consistent with the coverage data, the gene fragment amplified equally well in males and females, confirming that it is autosomal in this species, and therefore not the primary sex determinant. We similarly looked at the coverage of other scaffolds that had homology to known arthropod sex determination genes (table 1). All of the genes except for *virilizer* have significant similarity (E-value <10^−5^ with Blastx) to *A. franciscana* scaffolds, but these scaffolds have very similar coverage in the male and female sample. This suggests that either the master sex-determining gene is entirely different from those known in other arthropods or that, as suggested by Li et al. (2017), a W-linked noncoding RNA regulates *Masc* (as it does in Lepidoptera).

**Table 1:** Genomic Location of Candidate Sex-Determining Genes

Gene Name	Scaffold	log_2_F:M Coverage	Chromosome Assignment
*Transformer-2*	scaffold10099_size26673	0.038	Autosome
*Fruitless*	scaffold10814_size24206	−0.133	Autosome
*Runt*	scaffold1531_size111831	−0.044	Autosome
*Intersex*	scaffold15462_size12844	−0.241	Autosome
*Extra-macrochaetae*	scaffold1715_size105855	−0.096	Autosome
*Hopscotch*	scaffold1822_size102856	−0.087	Autosome
*Sex-lethal*	scaffold2058_size97338	−0.144	Autosome
*Deadpan*	scaffold225_size211206	−0.091	Autosome
*Doublesex*	scaffold312_size194285	−0.08	Autosome
*Female-lethal-d*	scaffold5986_size47945	0	Autosome
*Dissatisfaction*	scaffold772_size148565	−0.133	Autosome
*Daughterless*	scaffold903_size138824	−0.138	Autosome
*Groucho*	scaffold92_size266168	−0.085	Autosome
*Virilizer*	No assignment	No assignment	No assignment

## Discussion

While ZW sex determination had been hypothesized in *Artemia* for a long time (Bowen 1963), it was not until the recent production of a linkage map that it was fully supported (De Vos et al. 2013). Although eight AFLP markers were female-linked, suggesting a sex-linked region of ∼0.2 cM (De Vos et al. 2013), the nature and extent of differentiation of the sex chromosomes was still unknown. Here, we confirm the presence of a ZW system, and identify for the first time a large number of Z-specific genes, showing that a significant portion of the W has stopped recombining and has fully differentiated from the Z.

Interestingly, we also find evidence that there may be a nonrecombining region of the W chromosome that has already accumulated some divergence from the Z, but is not differentiated enough to lead to different genomic coverage in male and female samples. Differences in mutation rate or selective pressure along the degenerating W chromosome could in theory lead to such heterogeneity in ZW-divergence. However, the detection of such differences over thousands of (mostly noncoding) base pairs is usually thought to be due to recombination being lost at different time points on different parts of the sex chromosomes (Bergero and Charlesworth 2009; Wright et al. 2016), that is, to the formation of younger evolutionary strata. Reduced expression in the heterogametic sex is often a feature of young sex chromosome strata, and has been interpreted as evidence of early degeneration of the nonrecombining sex-specific chromosome (Y or W; Zhouand Bachtrog 2012; Gammerdinger et al. 2014; Hough et al. 2014). No such reduction is apparent in either tissue in our data (supplementary fig. S5, Supplementary Material online), suggesting that the putative young W stratum (or strata) of *A. franciscana* has not degenerated yet.

Since males and females appear to have similar recombination rates (De Vos et al. 2013), this putative progressive loss of recombination at different time points likely involved the acquisition of mechanisms that suppress recombination on the W, such as the fixation of inversions surrounding the sex-determining region. This is consistent with the canonical model of sex chromosome evolution, which postulates that repression of recombination along the W should be progressively favored in the presence of female-beneficial sexually antagonistic mutations, creating “evolutionary strata” of different ages (Bergero and Charlesworth 2009; Wright et al. 2016). While evolutionary strata have been detected in a variety of sex chromosomes (Lahn and Page 1999; Bergero et al. 2007; Wang et al. 2012; Schultheiß et al. 2015; White et al. 2015), including some ZW pairs (Nam and Ellegren 2008; Vicoso et al. 2013; Wright et al. 2014; Zhou et al. 2014), they are for the most part diverged enough that understanding what drove their evolution in the first place is difficult. The likely presence of both an ancient and a very young stratum therefore make *Artemia* a prime organism in which to investigate the dynamics of loss of recombination in a ZW system. More generally, ZW systems have been relatively understudied because, with the exception of Lepidoptera, they are generally not as amenable to experimental studies as model organisms such as fruit flies or nematodes. With their short life cycle (∼1 month), ease to keep, and the recent application of molecular tools such as RNAi (Sagi et al. 2013; Li et al. 2017), *Artemia* have the potential to become a great model for understanding the biology and evolution of ZW chromosomes.

The detection of many Z-specific genes in the older stratum allowed us to probe their expression and test for the presence of mechanisms of dosage compensation. While common in XY species, mechanisms that balance Z expression and lead to a full equalization of expression between males and females have only been found in Lepidoptera (but see Picard et al. 2018 for evidence that a similar mechanism may be evolving on the Z chromosome of schistosome parasites). Birds, snakes, and flatfish all express Z-specific genes at higher levels in ZZ males than in ZW females. Here, we detect no reduction in expression of Z-specific genes in females relative to males of *Artemia*, consistent with a mechanism of dosage compensation that affects the whole Z-specific region. There has been ample discussion of why ZW and XY pairs may differ in the extent to which they compensate their sex chromosome (recently reviewed in Gu and Walters 2017). Given that both Lepidoptera and *Artemia* have fully equalized expression of Z-specific genes, it appears that this difference may not hold for arthropods. Why this should be is still unclear. However, many hypotheses for why the Z may not become fully compensated base themselves on the reduced effective population size of the Z, which is greatly decreased by sexual selection on males. This reduced effective population size may make selection too inefficient for mutations that upregulate the expression of Z-linked genes to be fixed (Mullon et al. 2015; Gu and Walters 2017). Since arthropods generally have much larger effective population sizes than vertebrates, this reduction may not cause as dramatic a loss in the efficacy of selection (Huylmans et al. 2017). This discrepancy between vertebrates and invertebrates emphasizes the need to address evolutionary questions using a broad phylogenetic framework; future studies in other ZW groups with variable population sizes will be needed to test this and other hypotheses.

## Supplementary Material

Supplementary data are available at *Genome Biology and Evolution* online.

## Supplementary Material

Supplementary Data

## Notes

**Data deposition:** This project has been deposited at the NCBI Short Reads Archive under the accession PRJNA524488.
