# Supplementary material for: Sex-Biased Gene Expression and Dosage Compensation on the Artemia franciscana Z-Chromosome
Source: Genome Biol Evol. 2019 Mar 13;11(4):1033–44. doi: 10.1093/gbe/evz053 (PMC6456005; doi:10.1093/gbe/evz053)
Supplement: Supplementary Data [file evz053_supp.zip › Supplementary_Figures_and_tables.pdf]

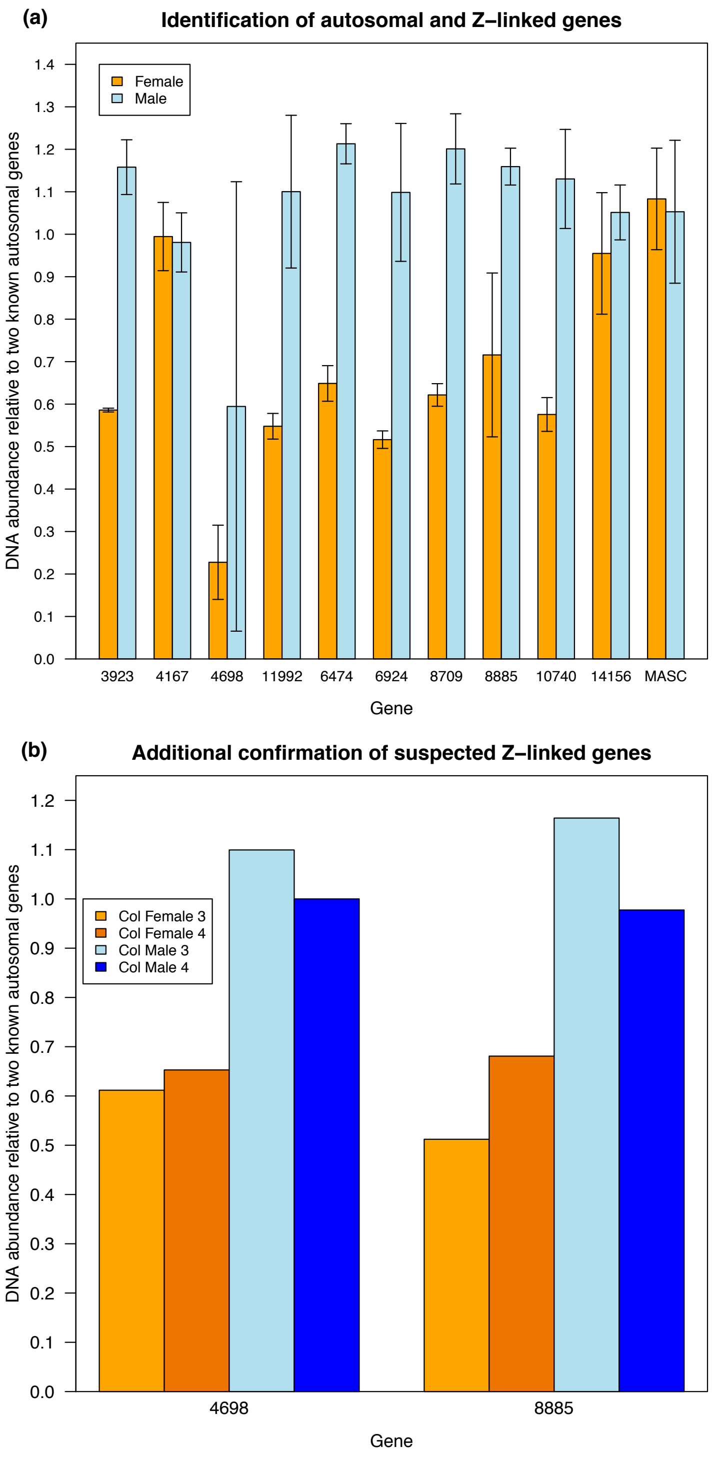


**Supplementary Figure 1:** qPCR validation of Z-specific scaffolds. A) Normalized DNA abundance for 10 putative Z-derived sequences and *Masc*, averaged for 3 males and 3 females. B) Amplification in two further males and two further males of Art-4698 and Art-8885, confirming they Z-linkage.

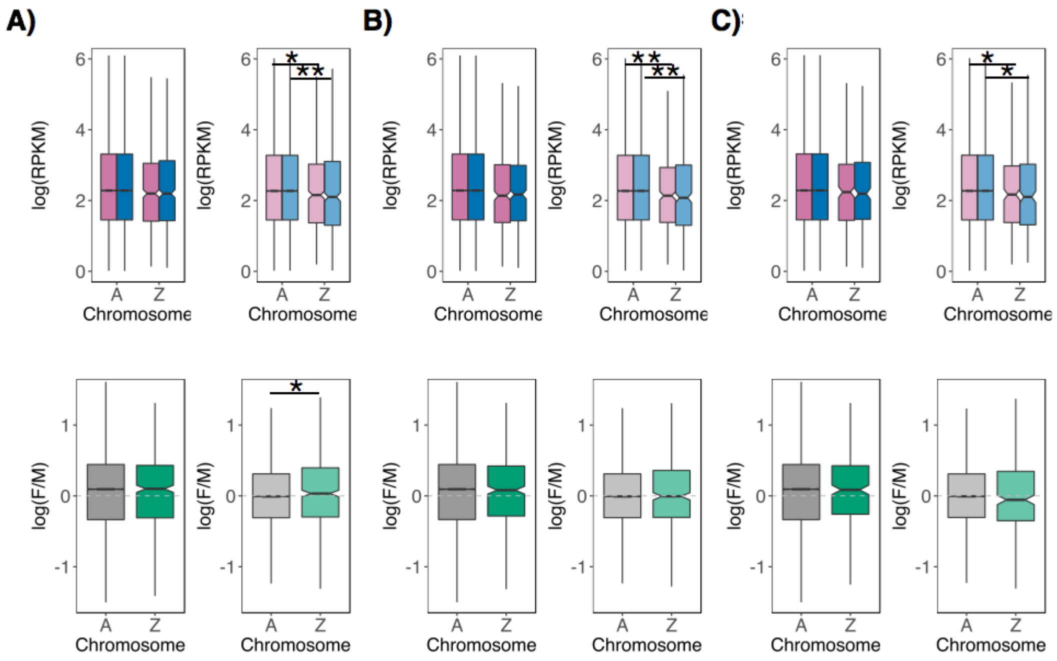


**Supplementary Figure 2:** Dosage compensation analysis for strict Z chromosome and autosomal thresholds. In the top row of all panels, gene expression of males (blue) and females (pink) on the autosomes ("A") and the Z chromosome ("Z") in gonads (left) and heads (right). In the bottom row of all panels, female-over-female ("F/M") expression ratios compared for the autosomes ("A", grey) and the Z chromosome ("Z", green) in gonads (left) and heads (right). A) Dosage compensation threshold 1 (median – 2 <= Z <= median – 0.6; median – 0.4 <=A <= median + 2). B) Dosage compensation threshold 2 (median – 2 <= Z <= median – 0.7; median – 0.3 <= A <= median + 2). C) Dosage compensation threshold 3 (median – 2 <= Z <= median – 0.8; median – 0.2 <= A <= median + 2). Wilcoxon test, *P-value<0.05, **P-value<0.01, ***P-value<0.001.

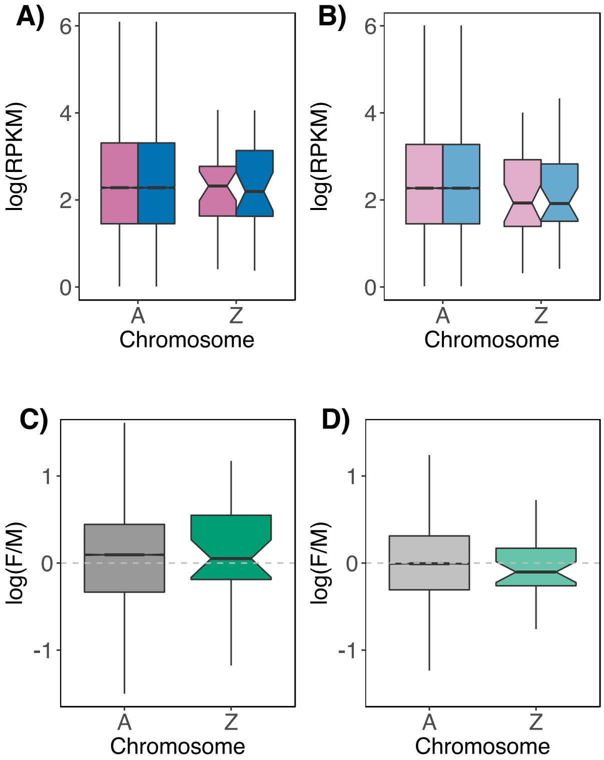


**Supplementary Figure 3:** Dosage compensation only for transcripts on qPCR-confirmed Z-linked scaffolds. A) Gene expression of males (blue) and females (pink) on the autosomes ("A") and the Z chromosome ("Z") in gonads. B) Gene expression of males (blue) and females (pink) on the autosomes ("A") and the Z chromosome ("Z") in heads. C) Female-over-male ("F/M") expression ratios compared for the autosomes ("A", grey) and the Z chromosome ("Z", green) in gonads. D) Female-over-male ("F/M") expression ratios compared for the autosomes ("A", grey) and the Z chromosome ("Z", green) in heads. Wilcoxon test, *P-value<0.05, **P-value<0.01, ***P-value<0.001.

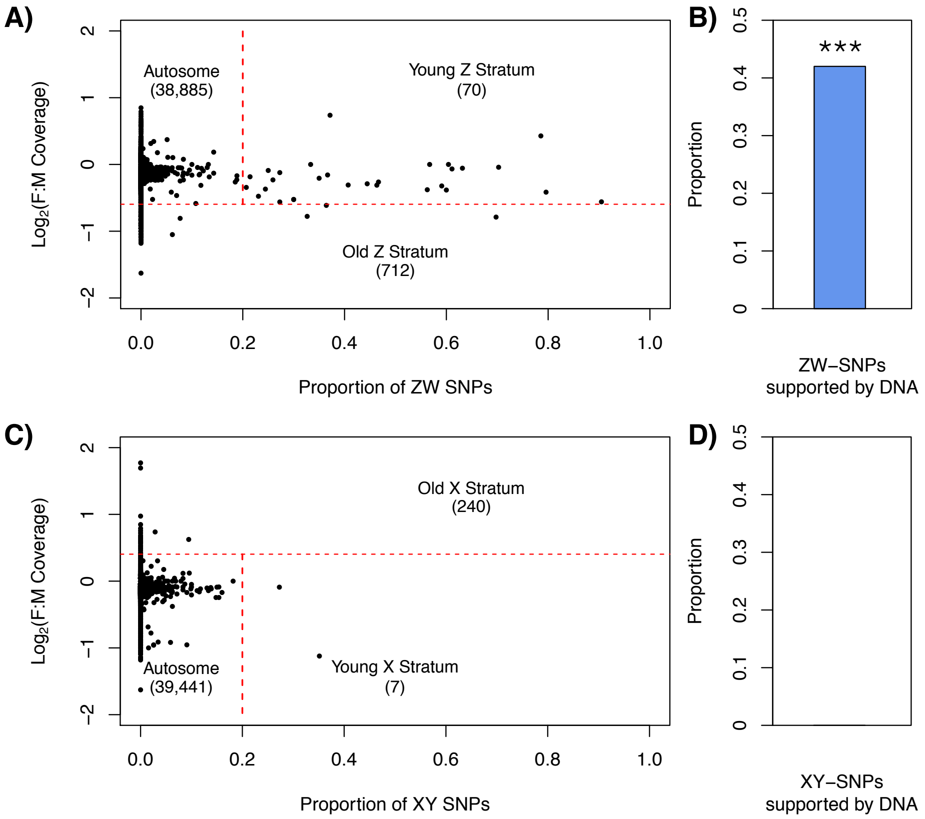


**Supplementary Figure 4: Identification of young Z stratum and false positive analysis of the young X stratum.** (A) Coverage and proportion of ZW SNPs on each scaffold. Scaffolds below the horizontal red dashed line are defined as Z-specific based on their $\log_2$(female:male) coverage ratio. Autosomal scaffolds are above the horizontal red dashed line and to the left of the vertical red dashed line. Young Z scaffolds are above the horizontal red dashed line and to the right of the vertical dashed line, and are defined by having greater than 20% of the SNPs consistent with ZW-inheritance. The number of transcripts in each category is given in parentheses. (B) The proportion of sex-chromosome consistent SNPs in the RNA data that are also consistent in the DNA sequencing dataset. Only SNPs on scaffolds with >20% of SNPs supporting a ZW karyotype in the RNA dataset (putative young ZW strata) were considered. (C) Coverage and proportion of XY SNPs on each scaffold. Scaffolds above the horizontal red dashed line are defined

as Z-specific based on their $\log_2$(female:male) coverage ratio. Autosomal scaffolds are below the horizontal red dashed line and to the left of the vertical red dashed line. Putative young X scaffolds are above the horizontal red dashed line and to the right of the vertical dashed line, and are defined by having greater than 20% of the SNPs consistent with XY-inheritance. The number of transcripts in each category is given in parentheses. (D) The proportion of sex-chromosome consistent SNPs in the RNA data that are also consistent in the DNA sequencing dataset. Only SNPs on scaffolds with >20% of SNPs supporting a XY karyotype in the RNA dataset (putative young XY strata) were considered. None were identified.

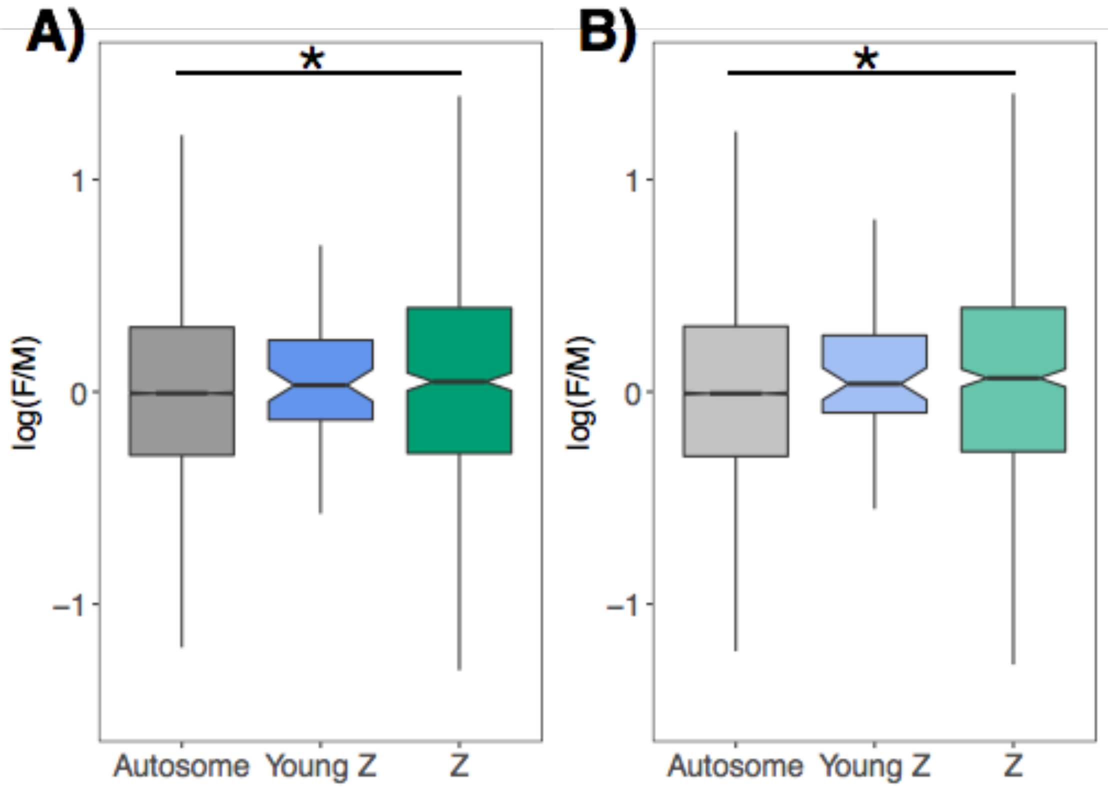


**Supplementary Figure 5:** Female-over-male ("F/M") expression ratios compared for the autosomes, the differentiated region of the Z chromosome, and the young Z stratum (A) in gonads and (B) in heads. Wilcoxon test, *P-value<0.05.

**Supplementary Table 1**. DNA profile of SNPs detected in the RNA dataset. The number of RNA SNPs that are invariant or polymorphic in the DNA dataset are shown for: 1. SNPs that were ZW-consistent in the RNA. 2. All SNPs detected in the RNA. ZW-consistent SNPs refer to SNPs that were heterozygous in the female but homozygous in the male, while SNPs showing the inverse pattern are called XY-consistent. SNPs that were ZW-consistent in the RNA dataset are strongly enriched in the DNA ZW-consistent category compared to other RNA SNPs ($P < 2.2 \times 10^{-16}$).

| In DNA | ZW SNPs RNA | All SNPs RNA | Proportion ZW RNA | Proportion all RNA |
|---|---|---|---|---|
| Invariant | 36 | 124,770 | 32.73% | 85.93% |
| Both heterozygotic | 3 | 5,487 | 2.73% | 3.78% |
| Fixed for different alleles | 22 | 2,790 | 20.00% | 1.92% |
| XY-consistent | 3 | 6,992 | 2.73% | 4.82% |
| ZW-consistent | 46 | 5,165 | 41.82% | 3.56% |

**Supplementary Table S2:** Scaffolds and genes used for the qPCR validation, as well as their respective primer sequences. *Art-11161* and *Art-15564* were used as autosomal controls, and *Masc* was putatively autosomal as well. All other genes were putatively Z-linked based on their coverage patterns.

| Scaffold | Gene | Male coverage | Female coverage | Log2(F/M) | Forward Primer | Reverse Primer |
|---|---|---|---|---|---|---|
| scaffold176_size224936 | Art-04167 | 26 | 17 | -0.6129769 | cggtccccttgtaaaactgg | caccttgcatgagtctcacg |
| scaffold467_size175125 | Art-08885 | 39 | 22 | -0.8259706 | caagacggaaattggagcca | accctcaaccgtttcattgc |
| scaffold548_size166836 | Art-08709 | 35 | 17 | -1.04182 | ctctttcaacggtgtctggc | aagtccatccgaagctagca |
| scaffold507_size171001 | Art-04698 | 34 | 18 | -0.9175378 | tgaagttggtgcagtgtgtt | cagcgaatcgggatcaaaca |
| scaffold863_size141797 | Art-10740 | 35 | 20 | -0.8073549 | aggggaatgccttagaacgg | gctctttcgttccactgcaa |
| scaffold9263_size30000 | Art-14156 | 36 | 19 | -0.9219975 | tgcgaaacaaaatagggaacaa | catcgacgtcttctttcccc |
| scaffold161_size232159 | Art-03923 | 35 | 19 | -0.8813555 | gagcccaccgaaaatacagc | aatctggctctgactgggtc |
| scaffold2360_size90732 | Art-06474 | 37 | 21 | -0.8171359 | ctggagcacgaattggagc | acatcaacaccaggacgatct |
| scaffold2388_size90221 | Art-11992 | 34 | 19 | -0.8395353 | ggcggtattatttgcttgga | gatgcacgaatggaaatgtg |
| scaffold2782_size83182 | Art-06924 | 31 | 16 | -0.9541963 | ccggatgatgatcgagaagt | tcttgtgtccagaagggtca |
| scaffold19_size413748 | Art-11161 | 35 | 33 | -0.0848889 | gctggtgcagagattgatga | ctgacggaaacattggttga |
| scaffold22_size400145 | Art-15564 | 36 | 33 | -0.1255309 | ggaaagaaaggtggccataa | tagaatgctttcggcaaacc |
| scaffold169_size230565 | MASC | 33 | 31 | -0.09019781 | aggaggttgcagcagtgttt | ctcttcctcaagtggcaacc |
